# Development of an Implementation Process Model: a Delphi study

**DOI:** 10.1186/s12913-021-06501-5

**Published:** 2021-06-07

**Authors:** Gillian Parker, Monika Kastner, Karen Born, Whitney Berta

**Affiliations:** 1 Institute for Health Policy, Management and Evaluation, University of Toronto, 155 College Street, 4th Floor, Toronto, ON M5T 3M6 Canada; 2 North York General Hospital, Centre for Research and Innovation, 4001 Leslie Street, Toronto, Ontario M2K 1E1 Canada

**Keywords:** Delphi, Implementation, Process, Model, Evidence-based, Health services research

## Abstract

**Background:**

There is general scarcity of research on key elements of implementation processes and the factors which impact implementation success. Implementation of healthcare interventions is a complex process. Tools to support implementation can facilitate this process and improve effectiveness of the interventions and clinical outcomes. Understanding the impact of implementation support tools is a critical aspect of this process. The objective of this study was to solicit knowledge and agreement from relevant implementation science and knowledge translation healthcare experts in order to develop a process model of key elements in the implementation process.

**Methods:**

A two round, modified Delphi study involving international experts in knowledge translation and implementation (researchers, scientists, professors, decision-makers) was conducted. Participants rated and commented on all aspects of the process model, including the organization, content, scope, and structure. Delphi questions rated at 75% agreement or lower were reviewed and revised. Qualitative comments supported the restructuring and refinement. A second-round survey followed the same process as Round 1.

**Results:**

Fifty-four experts participated in Round 1, and 32 experts participated in Round 2. Twelve percent (*n* = 6) of the Round 1 questions did not reach agreement. Key themes for revision and refinement were: stakeholder engagement throughout the process, iterative nature of the implementation process; importance of context; and importance of using guiding theories or frameworks. The process model was revised and refined based on the quantitative and qualitative data and reassessed by the experts in Round 2. Agreement was achieved on all items in Round 2 and the Delphi concluded. Additional feedback was obtained regarding terminology, target users and definition of the implementation process.

**Conclusions:**

High levels of agreement were attained for all sub-domains, elements, and sub-elements of the Implementation Process Model. This model will be used to develop an Implementation Support Tool to be used by healthcare providers to facilitate effective implementation and improved clinical outcomes.

## Background

Healthcare and the healthcare systems are constantly changing to incorporate new knowledge and evidence to improve health outcomes, patient experiences, system and process efficiencies, waste reduction and work experiences. Changing these processes through practice change interventions is a complex task.

A goal of implementation science is to understand factors that determine why an evidence-based intervention may or may not be successful in a specific healthcare setting and this information can be used to develop and test strategies to improve the speed, quantity and quality of uptake [1, 2]. A key area of implementation science is implementation support. Implementation support, such as using tools, training, and facilitation, have been shown to improve implementation processes and support better intervention outcomes [3].

Although literature on implementation support exists [2–6] there is little consensus on the key elements of the implementation process that are essential to successful implementation. Identifying these elements will be valuable to healthcare providers actively implementing healthcare interventions. This study endeavoured to refine key elements of implementation processes, concluding with an Implementation Process Model upon which the development of Implementation Support Tools can be based.

### Building on previous work

Our work was predicated on extensive work done by Kastner and colleagues on developing evidence-based, user-friendly knowledge translation (KT) and implementation support resources. In 2018, Kastner and colleagues produced and evaluated the *Knowledge-activated Tools (KaT) Framework* with the goal to detail steps and processes to support optimized, rigorous and efficient development of KT strategies [6]. Subsequently a Conceptual Implementation and Sustainability Guide (CISG) was drafted from the implementation and sustainability domains of the *KaT Framework* [6]. The process model upon which we focus in the current study was developed from elements of the CISG.

### Study objectives

The objective of this study was to refine and obtain agreement on an implementation process model via feedback from relevant experts in healthcare knowledge translation and implementation. This model can be used to inform the development of implementation support tools for healthcare interventions. The study sought expert agreement for four aspects of the implementation process model: 1) operationalized domains, subdomains and elements; 2) structure and order; 3) labels/terminology; and 4) applicability to target users.

## Methods

A Delphi process was used to refine and reach agreement on the key elements in healthcare implementation processes. Using a published framework as the basis for the study supported the goal of developing an evidence-based process model. The Delphi process consisted of two iterative rounds of ratings using an online survey. Aggregated results were distributed to participants after each round.

### Rationale for Delphi approach

The classical Delphi method is an iterative approach used to solicit and distill the judgments of experts using a series of surveys and feedback [7]. This process narrows the wide range of answers and serves to converge the group answers until consensus is reached [8]. Delphi studies are particularly effective in investigating areas where empirical data are lacking [9] and where priority setting is desired [10].

### Recruitment

A purposive sampling strategy was used to recruit a panel of international implementation science and knowledge translation experts. We updated the recruitment list produced for the *KaT Framework* Delphi study [6] to identify participants. This list included KT experts known or suggested by their project team; publicly available lists of individuals who have presented at implementation science, KT and health services research conferences and meetings (e.g., KT Canada, Alberta SPOR KT Platform, KT Connects, KT Scientific Meeting, Annual Science of Dissemination and Implementation conference, and Canadian Association for Health Services and Policy Research [CAHSPR]) and KT experts identified by other potential participants (snowball sampling). For the purpose of this study, expertise was defined as having knowledge or experience in KT or implementation science with the capacity to articulate informed opinion and provide relevant input about their area of expertise [10].

The recruitment strategy used email invitations containing a short description of the study, participation requirements, expectations of the participants, a request for referral for additional participants (snowball sampling) and a link to the online survey. We used an implied consent strategy whereby participants were informed that completion of the first survey was considered consent to participate in the study. Research and ethics board approval was obtained from the University of Toronto in August 2019.

### Inclusion criteria

The following eligibility criteria was developed to ensure the inclusion of international experts who have experience in KT or implementation science and knowledge or experience in developing and using active interventions, such as data feedback, communications training and systems-level interventions, in a healthcare setting. Inclusion criteria: 1) academic, researcher or healthcare practitioner with experience in these areas; and/or have published in these areas in the last 5 years; 2) sufficient written English skills to contribute relevant input and communicate ideas effectively; and 3) willingness and availability to complete up to three rounds of online surveys.

### Sample

Research suggests that a minimum panel of 15–20 experts is recommended to ensure sufficient contributions in a Delphi [11]. Taking account of the commonly high drop-out rate in Delphi studies, the recruitment target for this study was set at 30–40 participants for Round 1. This number would allow for the input of diverse views while accounting for expected attrition.

### Data collection

The Delphi study was conducted online over a four-month period to provide sufficient time to gather data, aggregate and communicate group responses, and to build surveys step-wise as data were collected and analyzed. The surveys were developed and designed using *Survey Monkey,* an online survey platform (www.surveymonkey.com). Prior to administration, the first survey was pre-tested by two volunteers for clarity and to anticipate the average completion time. The survey was revised as a result of the pre-test. A link to each survey was distributed via email to all participants with subsequent follow up emails as necessary. Data collection took place between October 2019 and January 2020.

### Round 1 survey

The first survey was comprised of 5-point Likert scale questions with comments and free-text questions. The purpose of this round was to invite participants to: 1) rate the importance of the content and structure of the process model; 2) suggest additional elements/concepts they deemed important to the implementation process; and 3) recommend items to be removed from the process model.

The first-round survey also collected the following demographic information: age; gender; primary role; years of experience with KT science/practice, implementation, Integrated KT, dissemination and de-implementation; and years of experience with developing a KT framework or model and experience with implementing a KT framework or model. The process model was modified and refined based on the percent agreement rating and qualitative data.

After Round 1, participants received a summary of the results including questions which reached or did not reach agreement, and descriptive statistics, including the mean, standard deviation, median, interquartile range and percent agreement for all questions. Consensus to include an item was defined as a mean score of 4 out of 5 on a Likert scale (1 = Strongly Disagree, 2 = Disagree, 3 = Neither Agree or Disagree, 4 = Agree, 5 = Strongly Agree) by greater than 75% of Delphi participants. Participants also received, via email, table of substantive changes made to the process model based on the Round 1 results and the refined version of the process model to review in advance of the Round 2 survey.

### Round 2 survey

The Round 2 survey asked participants to review and rate the revisions made to the content and organization of the process model. Participants were also asked again to rate the comprehensiveness of the implementation process elements. All questions provided the opportunity to provide comments or feedback. The second survey was designed to 1) determine agreement on items revised based on results of Round 1; and 2) determine preliminary agreement of the new items generated in Round 1; and 3) elicit further comments and feedback. The participants were asked again to rate the questions using a 5-point Likert scale and use the free text sections to state the reasoning for their rating or provide additional comments.

After Round 2, participants received a summary of the results including descriptive statistics for all questions. Consensus was defined as higher than 75% agreement on a question. Participants also received a copy of the final Implementation Process Model.

### Data analysis

#### Quantitative

Results were tabulated at the completion of each round and entered into an Excel spreadsheet. Descriptive statistics – mean, median, inter-quartile range (IQR), standard deviation, and percent agreement – for each question were reported for Round 1 and Round 2 results. Participants received the summary Round 1 results in advance of the Round 2 survey and were free to review and reflect on these results as they submit their responses and feedback in Round 2.

#### Qualitative

The data were analysed by using thematic analysis [12]. Following Braun & Clarke (2006), initially the participant comments were read, and re-read to gain familiarity. Subsequently, words, phrases and sentences were coded and organized into themes [12]. Then themes were reviewed in relation to coded sections and themes were refined [12]. Codes and themes were reviewed independently by two team members (GP, MK) to cross-check data analysis and ensure data quality, consistency in approach and transparency of analytical decision making. Differences in interpretation were resolved through discussion between the researchers.

## Results

### Participant characteristics

**Fig. 1:**
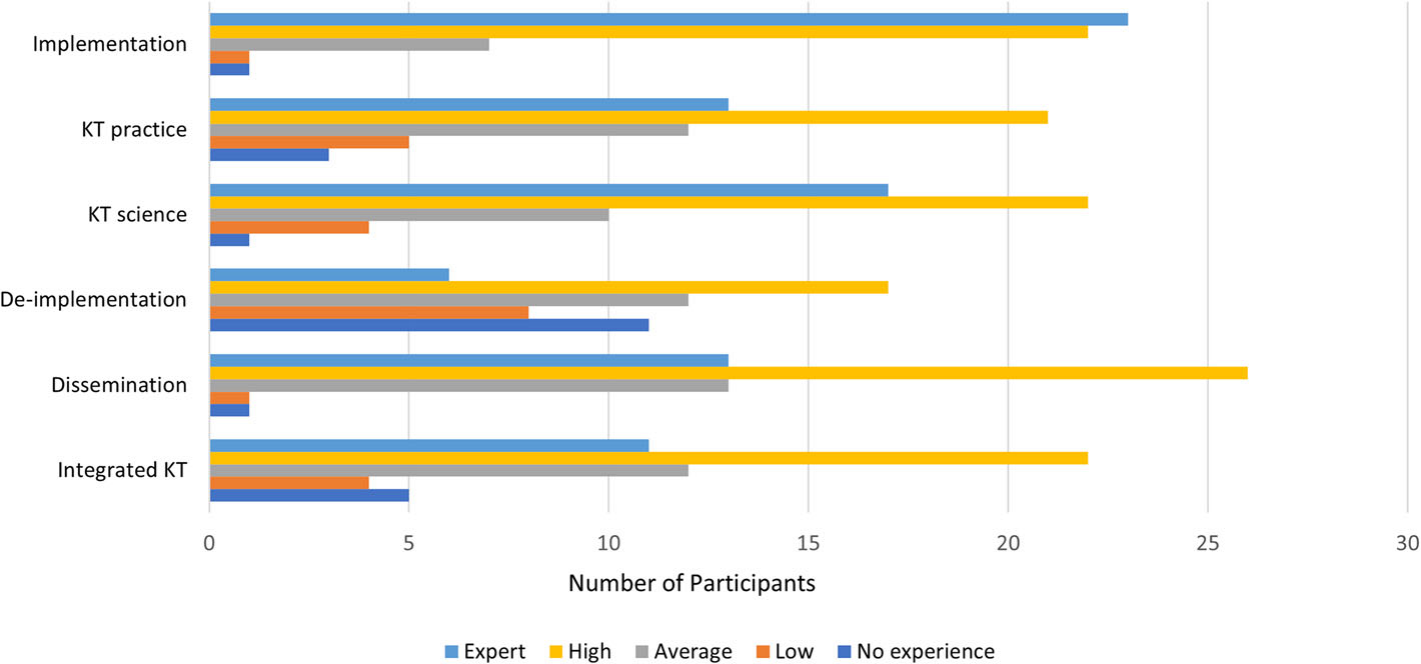
Participant Experience

Five hundred and thirty-four survey links were sent via email, 88 viewed the survey and 54 experts (10%) participated in the Round 1 survey. The characteristics of participants are shown in Table 1. The majority of participants were women (59%) in the 55 to 64 age range (30%). The majority of participants (83%) were Researchers, Scientists or Professors living in Canada (41%), United States (39%) and the United Kingdom (7.4%). The majority of participants rated their experience with Implementation (83%), KT Science (72%), Dissemination (72%), KT practice (63%) and Integrated KT (61%) as high or expert. De-implementation expertise was rated as high or expert by 23 participants (43%) (See Fig. 1).

**Table 1:** Round 1: Participant Characteristics

Characteristic	Round 1(*n* = 54)
**Age**	
18 to 24	0 (0%)
25 to 34	3 (5.56%)
35 to 44	15 (27.78%)
45 to 54	11 (20.37%)
55 to 64	16 (29.63%)
65 to 74	9 (16.67%)
75 or older	0 (0%)
**Gender**	
Female	32 (59.26%)
Male	22 (40.74%)
Other	0 (0%)
Prefer not to say	0 (0%)
**Primary Role**	
Researcher/Scientist/Professor	45 (83.33%)
Clinician	2 (3.70%)
Policymaker	1 (1.85%)
Other Decision-maker	2 (3.70%)
Trainee	1 (1.85%)
Other	3 (5.56%)
**Location**	
United States	21(38.89%)
Canada	22(40.74%)
United Kingdom	4(7.41%)
Australia	3(5.56%)
Norway	2(3.70%)
Germany	1(1.85%)
Netherlands	1(1.85%)

### Round 1

Survey questions pertained to the content of the elements, order of the elements and comprehensiveness of the sub-domains. In Round 1 participants reached agreement for 46 of the 52 content questions. The questions which did not reach agreement concerned the comprehensiveness of the Element (*n* = 4) (Engaging Stakeholders, Monitoring and Evaluation) and the order of elements within a sub-domain (*n* = 2) (Developing the Implementation and Sustainability Plan, Monitoring and Evaluation). Mean scores ranged from 3.4 to 4.4, with the standard deviation ranging from 1.2 to 0.5. Table 2 provides the results for Round 1, including the mean, standard deviation, median, IQR, percent agreement. Questions that reached greater than 75% agreement were included unless qualitative data was contrary and reached agreement to amend the item.

**Table 2:** Round 1: Results

Implementation and Sustainability Sub-domains, Elements and Sub-elements	N	Mean	SD	Median	IQR	% Agree
***Implementation and Sustainability Sub-Domains***						
*These four sub-domains appropriately represent necessary steps in the implementation and sustainability process of healthcare innovations.*	**54**	**3.8**	**0.9**	**4**	**0**	**75%**
1. Engage relevant stakeholders and establish partnerships to:						
2. Identify the implementability and sustainability of the innovation						
3. Develop an implementation and sustainability plan						
4. Monitor and evaluate the implementation and sustainability of the intervention/innovation						
*The order of the four sub-domains makes sense.*	54	4.0	1.0	4	1	80%
*The components of the sub-domains are comprehensive.*	**54**	**3.4**	**1.2**	**4**	**2**	**68%**
***1. Engage relevant stakeholders and establish partnerships to:***						
*The following elements are necessary for this sub domain:*						
a. Determine the objectives and goals for implementation	54	4.4	0.8	5	1	89%
b. Determine optimized communication mechanism among team	54	4.1	0.8	4	1	82%
c. Identify and clarify roles	54	4.2	0.8	4	1	85%
d. Identify any anticipated challenges and mitigating strategies to implementation	54	4.2	0.8	4	1	84%
The order of the four elements makes sense.	54	3.9	0.9	4	0	78%
The components of the elements are comprehensive.	**54**	**3.4**	**1.2**	**4**	**2**	**67%**
***2. Identify the implementability and sustainability of the innovation***						
*The following elements are necessary for this sub domain:*						
a. Identify the purpose of the implementation and sustainability	54	4.0	0.9	4	1	80%
b. Assess the determinants of implementation and sustainability *(barriers and facilitators to change)*	54	4.4	0.6	4	1	89%
c. Assess Readiness to Change	54	4.3	0.6	4	1	86%
The order of the three elements makes sense.	54	4.0	0.9	4	1	79%
The components of the elements are comprehensive.	54	3.8	0.9	4	1	76%
***3. Develop an implementation and sustainability plan***						
*The following elements are necessary for this sub domain:*						
a. Identify stakeholders who should be involved in the implementation	54	4.4	0.6	4	1	88%
b. Assess the context and characteristics of the adopter environment	54	4.3	0.7	4	1	87%
c. Assess the fit and effectiveness of the intervention/innovation	54	4.1	0.9	4	1	82%
d. Assess fidelity and adaptation of the intervention/innovation	54	4.1	0.9	4	1	82%
e. Assess the capacity to sustain the intervention/innovation	54	4.3	0.6	4	1	86%
f. Adapt learnings from implementation	54	4.2	0.8	4	1	83%
g. Consider the use of an implementation framework	54	4.1	0.8	4	1	82%
h. Consider the use of a sustainability framework	54	4.1	0.8	4	1	81%
The order of the eight elements makes sense.	**54**	**3.6**	**1.1**	**4**	**1**	**72%**
The components of the elements are comprehensive.	54	3.8	0.9	4	1	77%
***4. Monitor and evaluate the implementation and sustainability of the intervention/innovation***						
*The following elements are necessary for this sub domain:*						
a. Engage stakeholders and knowledge users	54	4.3	0.5	4	1	86%
b. Define the scope of implementation and sustainability	54	4.1	0.7	4	1	83%
c. Identify objectives and purpose of the evaluation	54	4.3	0.5	4	1	86%
d. Determine the focus of the implementation and sustainability evaluation	54	4.3	0.6	4	1	86%
The order of the above elements makes sense.	54	3.8	1.0	4	0	77%
The components of the elements are comprehensive.	**54**	**3.7**	**1.0**	**4**	**1**	**73%**
*The following element and sub-elements are necessary for this sub domain:*						
e. Select the type of evaluation	54	4.2	0.8	4	1	85%
Formative evaluation	54	4.1	0.7	4	1	83%
Implementation or process evaluation	54	4.3	0.7	4	1	86%
Outcome evaluation	54	4.3	0.7	4	1	86%
Economic evaluation	54	4.1	0.8	4	1	82%
Impact evaluation	54	4.1	0.7	4	1	82%
Summative evaluation	54	4.2	0.7	4	1	83%
Hybrid model	54	4.0	0.7	4	2	80%
**The order of the seven sub-elements makes sense.**	**54**	**3.8**	**0.9**	**4**	**1**	**75%**
The components of the sub-elements are comprehensive.	54	4.0	0.7	4	0	81%
*The following elements and sub-elements are necessary for this sub domain:*						
f. Select the appropriate study design(s) for the type of evaluation	54	4.2	0.7	4	1	83%
g. Select outcomes and establish indicators	54	4.4	0.6	4	1	87%
Clinical	54	4.3	0.8	4	1	85%
Patient	54	4.3	0.8	4	1	85%
Provider	54	4.3	0.7	4	1	86%
Process or implementation	54	4.3	0.7	4	1	86%
Service	54	4.2	0.7	4	1	84%
Organizational and health care system	54	4.2	0.7	4	1	85%
Economic	54	4.2	0.7	4	1	85%
h. Consider using implementation evaluation frameworks to drive the evaluation	54	4.1	0.8	4	1	82%
The order of the elements and sub-elements make sense.	54	3.8	0.9	4	1	76%
The components of the elements and sub-elements are comprehensive.	54	3.8	0.8	4	1	76%

Bold items did not reach consensus (< 76%)

IQR: 0 = high consensus; 1 = good consensus; 2 = poor consensus

Participants also made recommendations in the comments sections regarding items for addition or removal. Thematic analysis of the recommendations and feedback provided in the Round 1 survey identified themes to be addressed and incorporated into the revisions for Round 2. The key themes identified in the Round 1 survey:

### Stakeholder engagement

Participants emphasized that stakeholder engagement should not happen at a specific point in the process, but rather is critical throughout the planning, implementation, monitoring and evaluation processes. One Delphi participant commented: *“There is a ‘stream’ of stakeholder engagement work that cuts across all domains. Some of the work has a natural sequence and some might be done by different or the same stakeholders at roughly the same time period.”[P26].*

Participants noted that engagement should be integrated throughout and must also involve accountability and responsibility for all parties. A participant noted: *“There should be something added around ensuring meaningful engagement of stakeholder partners e.g., through building trusting relationships, valuing diverse expertise and knowledge, shared decision-making, shared goals, etc.”[P20]* To address the feedback received, the process model was refined, and *Engaging Stakeholders* was included throughout the three sub-domains.

### Context

The expert panel also felt that the importance of understanding, identifying and planning for the impact of context on the implementation process was underrepresented in the model. A number of participants stated that context and the actions which address it, need to be explicit in the process model: *“I don’t see how the issue of context is highlighted; it may be implicit, but in my view since implementation is a function of the intervention by context interaction, context and potential interactions should be explicit.”[P42]* In response to this feedback, context and the actions required to address it were explicitly added to elements of the process model. Context was made explicit in the first step of planning: *Identify the purpose of the Implementation and Sustainability of the intervention/innovation*. In addition, context was incorporated into 4 other elements where it was applicable.

### Implementation as an iterative process

Many participants discussed that implementation and sustainability are iterative non-linear processes. Participants acknowledged the need for logical presentation and helpful heuristics when documenting implementation in a process model but asked that the non-linearity of implementation be highlighted. A participant stated: “*… you need to be clear that these are steps to be covered, not steps to be followed. Iteration will often be necessary, and flexibility is required depending on the situation.”[P04].*

One participant emphasized the impact of non-linearity on implementation efforts: *“Planning allows us to prepare for contingencies, to form alliances, to gather resources. It allows us to articulate a clear statement of our intentions, and of the actions needed to achieve those intentions. However, when the plan is complete and action has begun, it is essential that we do not follow a rote, fixed implementation of the plan. Rather, we watch the plan as it unfolds, we notice what is working or not working, and we revise and adjust as we go. Each situation will be different, each social form will be characterized by unique affordances and constraints. We are firm in our intentions and flexible in our actions.”* [P51] The guidance for the process model was amended to explicitly acknowledge that implementation is an iterative process and that the elements detailed in the model represent evidence-based components to consider and address to support implementation, but do not require a sequential completion.

### Use of theories or frameworks

The value of using theory or frameworks to guide implementation was also highlighted by participants. One participant commented: *“One always uses a framework or mental model. The only question is whether it is made explicit. And it should be.”[P42]* Participants also discussed the importance of aligning theories or frameworks with the intervention. One participant noted: *“[This Element] should state that the framework must be matched to the problem and determinants.”* [P16] The selection of guiding frameworks was moved up in the model and additional guidance was added regarding selection and application of theories and frameworks.

### Amendments as a result of round 1

Nineteen changes were made to the process model based on the responses received in Round 1. Changes applied to location (*n* = 11), removal (*n* = 4) and addition (*n* = 4) of sub-domains/elements/sub-elements. These changes were reported in a table of substantive changes made to the process model and a refined version of the process model.

### Round 2

**Fig. 2:**
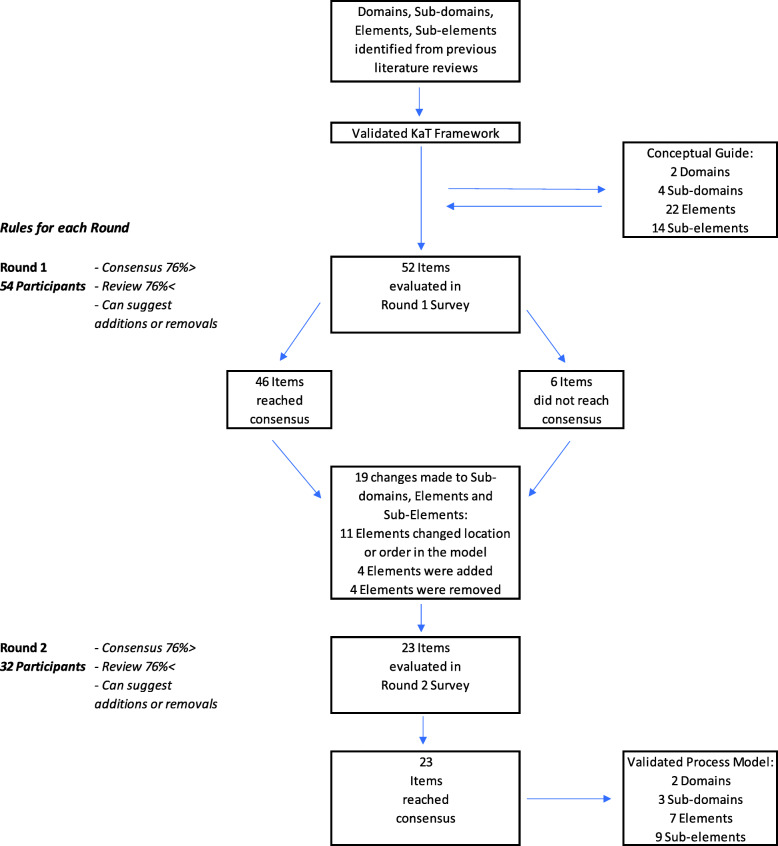
Summary of the Delphi Process

**Fig. 3:**
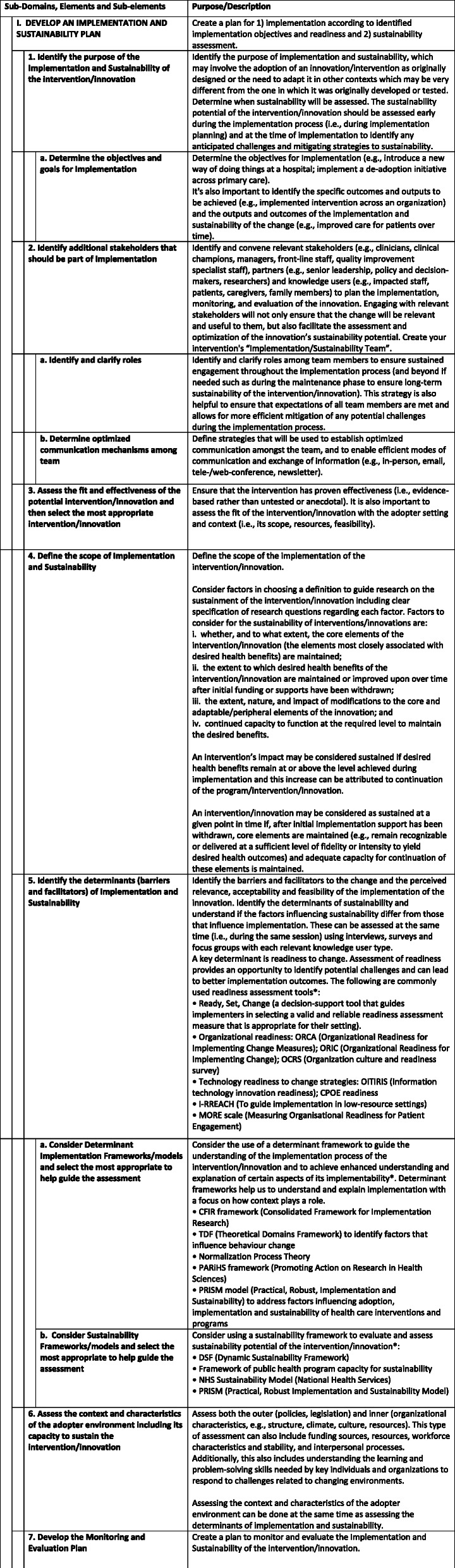
Implementation Process Model

For Round 2, 59% of Round 1 participants completed the survey (*n* = 32). The 19 amendments to the process model were represented in 23 survey questions which were evaluated by the participants. Again, participants were provided with comment sections on each question to provide additional feedback. All 23 questions reached agreement in the Round 2 survey. Participants provided additional feedback on the need for consistent terminology and also the need to further clarify the target user for the tool. Mean scores ranged from 3.8 to 4.8, with the standard deviation ranging from 1.0 to 0.3. Table 3 provides the results for the Round 2 Survey. Figure 2. illustrates the Delphi Process Summary. Figure 3. demonstrates the final, Implementation Process Model.

**Table 3:** Round 2: Results

Implementation and Sustainability Sub-domains, Elements and Sub-elements	N	Mean	SD	Median	IQR	% Agree
***Instructions/Guidance section***						
The above suggestions should be incorporated into the Instructions/ Guidance.	32	4.3	0.6	4	1	97%
***Engage Relevant Stakeholders***						
Engaging Relevant Stakeholders should occur throughout the process.	32	4.8	0.4	5	0	100%
***Sub-domain One: Develop an Implementation and Sustainability Plan***						
***Element 1***						
The ‘Content’ changes (tracked in blue) make sense.	32	4.0	0.9	4	0	84%
The order above makes sense.	32	4.1	0.7	4	0	97%
The components above are comprehensive.	32	3.9	0.8	4	0	81%
***Element 2***						
The ‘Content’ changes (tracked in blue) make sense.	32	4.3	0.7	4	1	94%
The order above makes sense.	32	4.2	0.5	4	0	97%
The components above are comprehensive.	32	4.0	0.6	4	0	84%
***Elements 3 & 4***						
The ‘Content’ changes (tracked in blue) make sense.	32	4.0	0.9	4	1	84%
The order above makes sense.	32	4.0	0.9	4	1	87%
The components above are comprehensive.	32	4.0	0.8	4	0	84%
***Elements 5 & 6***						
The ‘Content’ changes (tracked in blue) make sense.	32	3.8	1.0	4	0	84%
The order above makes sense.	32	3.8	1.0	4	0	77%
The components above are comprehensive.	32	3.9	1.0	4	0	81%
***Element 7 a & b***						
The ‘Content’ changes (tracked in blue) make sense.	32	4.1	0.7	4	0	94%
The order above makes sense.	32	4.3	0.5	4	1	97%
The components above are comprehensive.	32	3.9	0.9	4	0	81%
***Element 7 c & d***						
The ‘Content’ changes (tracked in blue) make sense.	32	4.0	0.8	4	0	84%
The order above makes sense.	32	4.0	0.7	4	0	90%
The components above are comprehensive.	32	3.8	1.0	4	0	77%
***Sub-domains Two & Three: Implementation and Monitor and Evaluate***						
The ‘Content’ changes (tracked in blue) make sense.	32	4.1	0.5	4	0	94%
The order above makes sense.	32	4.1	0.3	4	0	100%
The components above are comprehensive.	32	3.8	0.8	4	0	84%

IQR: 0 = high consensus; 1 = good consensus; 2 = poor consensus

## Discussion

We performed a rigorous, modified Delphi study involving an international panel of KT and implementation experts to organize, prioritize and evaluate the key elements of an implementation process model. This is the first study, to our knowledge, in which the domains, sub-domains, elements and sub-elements of intervention implementation processes were evaluated and refined to develop an implementation process model. Our findings confirm earlier work on identifying evidence-based elements in the complex process of implementing healthcare interventions [6].

### Summary of key findings

Stakeholder engagement was identified as a critical component of the implementation process. The CISG identified stakeholder engagement as the first sub-domain in the process and as a result of this Delphi, stakeholder engagement was integrated throughout the process model. Stakeholder engagement has been defined as “an iterative process of actively soliciting the knowledge, experience, judgment and values of individuals selected to represent a broad range of interests in a particular issue, for the dual purposes of creating a shared understanding and making relevant, transparent and effective decisions” [13]. Research states that effective stakeholder engagement supports effective study design, data analysis and research prioritization [14]. In addition, and potentially most significant, studies report that effective stakeholder engagement improved perceived relevance and uptake of research findings [15]. The notion that stakeholder engagement should be integrated throughout and involve accountability and responsibility for all parties is prevalent in the literature which reports that accountability should be interactive between researchers, practitioners and evaluators with shared goals to achieve results [4, 16].

Delphi participants emphasized the importance of context in the implementation process. The impact of context on the implementation process is well documented in the research, but as Dryden-Palmer et al. noted, a thorough understanding of how context modifies or impacts implementation is lacking [5]. The influence of context on implementation and the need to adapt or tailor interventions to context has been recognized as essential to implementation success. Context can be the environment, setting, or organizational structure and can act as either a barrier or facilitator to implementation [5]. Making the impact and importance of context explicit in the process model is important as healthcare providers who are implementing interventions need support and guidance when adapting interventions to new settings and environments [4].

Aligning with our expert participants, the literature supports that implementation is a dynamic process which does not unfold in a linear fashion [17]. As a result of the Delphi, our process model acknowledges that moving evidence into practice is complex and often unpredictable and is influenced by many factors [18].

Nilsen noted that research with underused or misused theoretical perspectives makes it difficult to understand and explain how and why interventions succeed or fail, “thus restraining opportunities to identify factors that predict the likelihood of implementation success and develop better strategies to achieve more successful implementation” [19]. The need for theory has also been documented in two recent reviews of systematic reviews of the effectiveness of single and multifaceted interventions to change provider behaviour [20, 21]. The authors advocated for more research to develop a theoretical base for intervention selection or development and for tailoring interventions, based on identified barriers and facilitators, to increase their effectiveness [20, 21]. In addition, the importance of aligning theories or frameworks with the intervention is noted in the literature [17]. Research has identified that clinical outcomes are improved when theories or frameworks guide the implementation process, with specific attention paid to the fit with context [22]. These sentiments were expressed by participants and the process model was amended to reflect the significance and value added by using theories and frameworks to guide implementation.

### Implications for policy and practice

Healthcare interventions are challenging to implement, and healthcare providers are often not experts in implementation and therefore need resources and support to succeed. Our findings offer a resource for providers and can inform tool development processes.

By evaluating and refining the elements in the implementation process we have developed an evidence-based foundation to create a simple, user friendly tool that will be effective to support both implementation effectiveness and improved clinical outcomes. The findings of this Delphi study confirm the results of previous work [6, 23] and underscore the importance of implementation support to facilitate effective, sustainable, improved outcomes for healthcare interventions.

### Strengths and limitations

Our Delphi study has several strengths. Our international panel was composed of KT and implementation science and practice experts, which helped to ensure a high level, yet diverse range of expertise contributed to the findings. Using this Delphi technique ensured more diversity in expertise than would be provided from any individual member or small related group. By engaging this diverse group, we have been able to increase the generalizability and creditability of the results.

By providing the opportunity for free-text responses we ensured that participants could offer context to their ratings where they felt it necessary or helpful. This design helped to explicate the rationale and perspectives of the experts. In addition, the free-text entries allowed participants to address items and topics they felt were missing from the process model.

The anonymity in a Delphi study is both a strength and limitation, it helps to reduce the influence of participants who may dominate an in-person session but also eliminates the opportunity for the discussion and discovery that can occur during in-person meetings. There may have been bias in the selection of elements presented to the participants. We minimized this through an extensive literature search and provided participants with the opportunity to add elements to the process model in the first and second Delphi rounds. The potential influence of local factors, such as culture, healthcare systems or policy on participant’s responses should be acknowledged. While the 10% response rate may limit the generalizability of our findings, the diversity and number of participants in our sample was representative. Finally, while we included clinicians in our invite list, the sample for our Delphi turned out to be largely academic and the process may have benefitted from additional participation by healthcare providers. We will be mindful of recruitment for the tool development project to ensure more healthcare providers participate as they will be the primary target users.

## Conclusions

The Delphi survey questions covered a comprehensive range of aspects of the implementation process from planning to identifying barriers to monitoring and evaluating. Using the Delphi process to gain agreement among a group of international experts, we produced an implementation process model which will be used to develop a user-friendly and evidence-based tool. This tool will be designed to support healthcare implementation efforts with the goal to improve process and clinical outcomes.

## Data Availability

The datasets supporting the conclusions of this article are included within the article and its additional files.

## Abbreviations

KT: Knowledge translation
KaT: Knowledge-activated Tools
CISG: Conceptual Implementation and Sustainability Guide
IQR: Interquartile range
